# Correction: Immunologic monitoring of cytomegalovirus (CMV) enzyme-linked immune absorbent spot (ELISPOT) for controlling clinically significant CMV infection in pediatric allogeneic hematopoietic stem cell transplant recipients

**DOI:** 10.1371/journal.pone.0294486

**Published:** 2023-11-13

**Authors:** Euri Seo, Eun Seok Choi, Jung Hwa Kim, Hyery Kim, Kyung-Nam Koh, Ho Joon Im, Jina Lee

There are errors in the Funding section. The correct Funding statement is: This study was supported by a grant from Asan Institute for Life Sciences (grant No. 2016–0220).
