# Supplementary figures and images for: Immunologic monitoring of cytomegalovirus (CMV) enzyme-linked immune absorbent spot (ELISPOT) for controlling clinically significant CMV infection in pediatric allogeneic hematopoietic stem cell transplant recipients
Source: PLoS One. 2021 Feb 5;16(2):e0246191. doi: 10.1371/journal.pone.0246191 (PMC7864450; doi:10.1371/journal.pone.0246191)

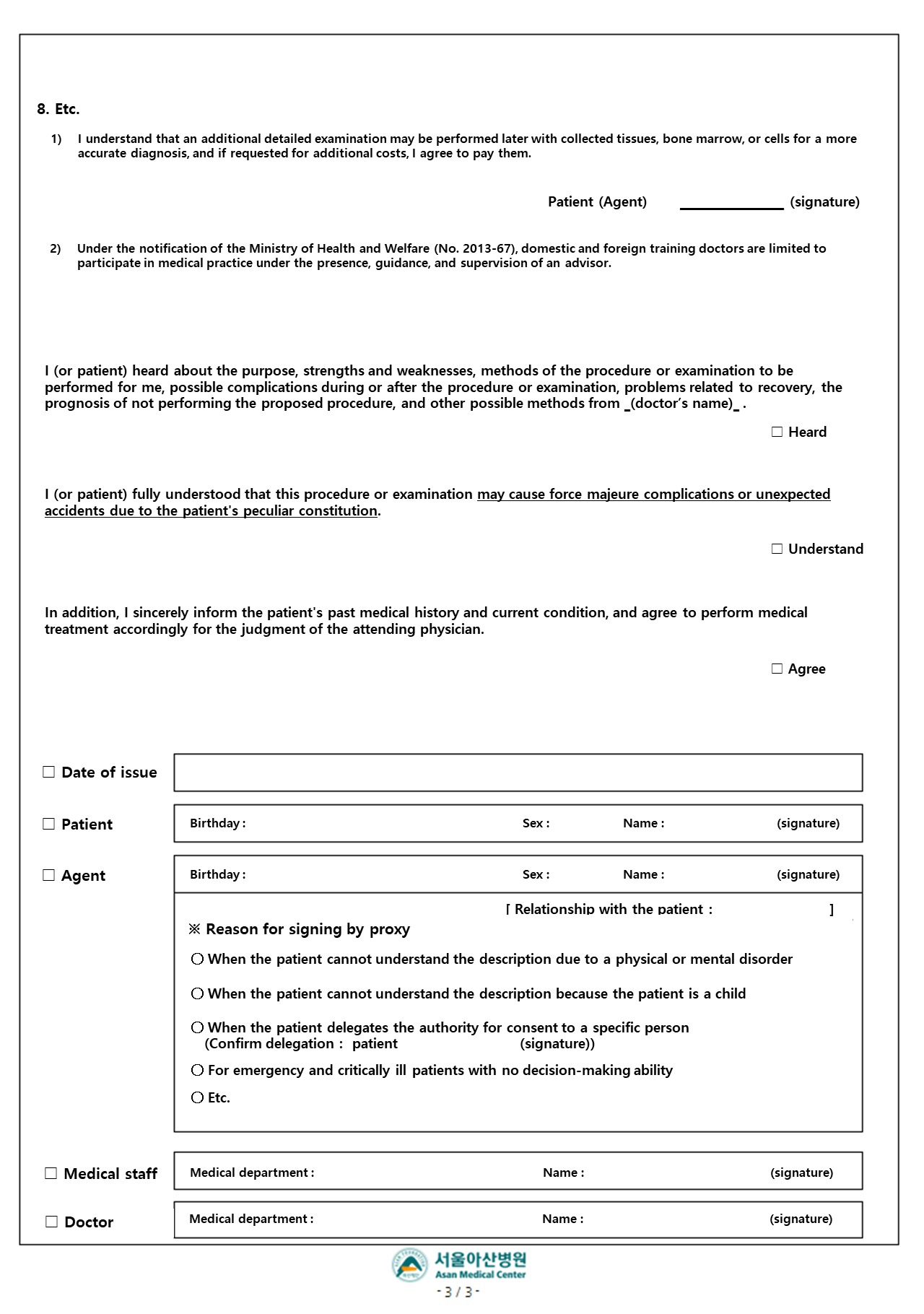

Supplement: S1 Data — (ZIP) [file pone.0246191.s001.zip › ConsentfromDonor3E.png]

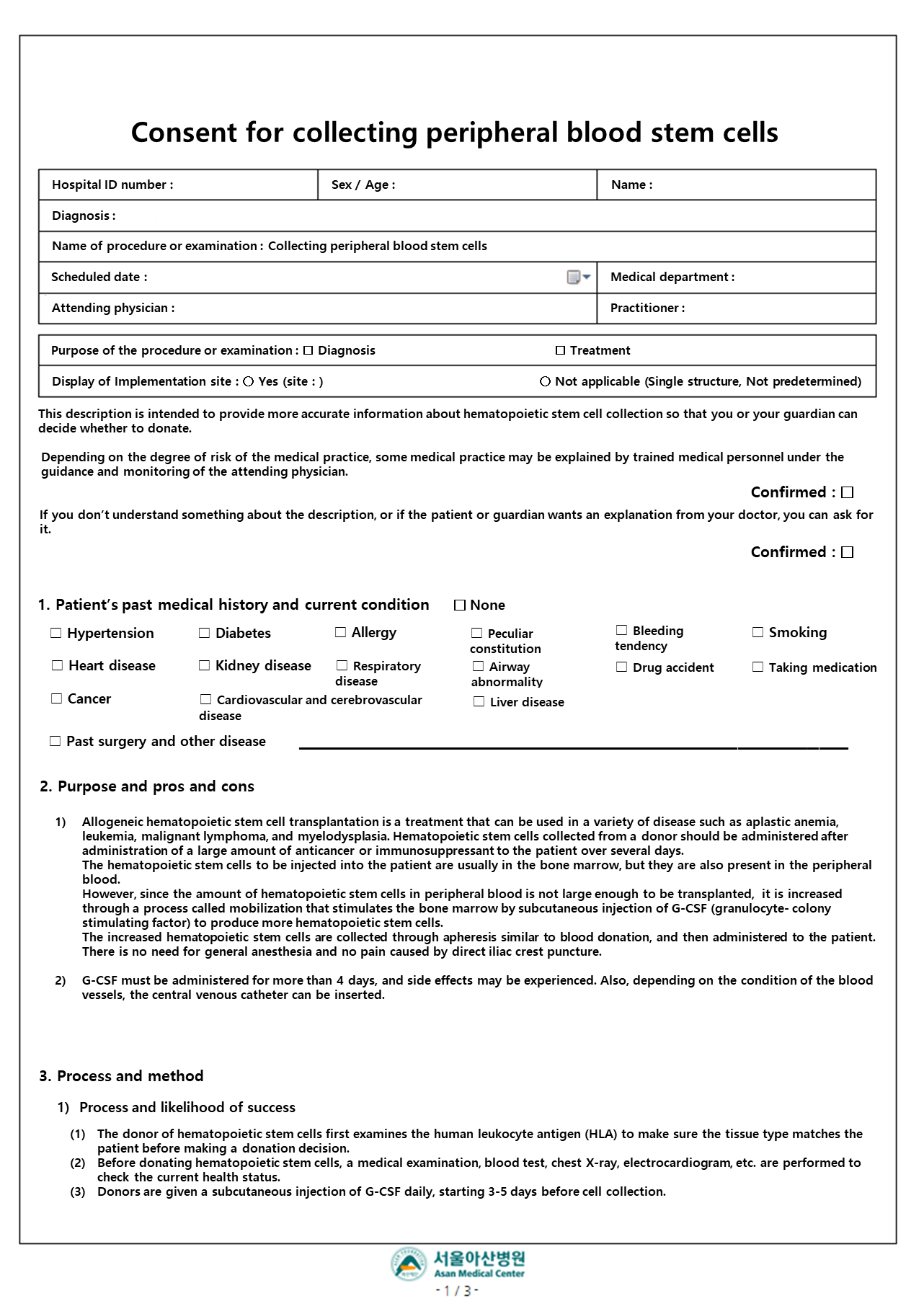

Supplement: S1 Data — (ZIP) [file pone.0246191.s001.zip › ConsentfromDonor1E.png]

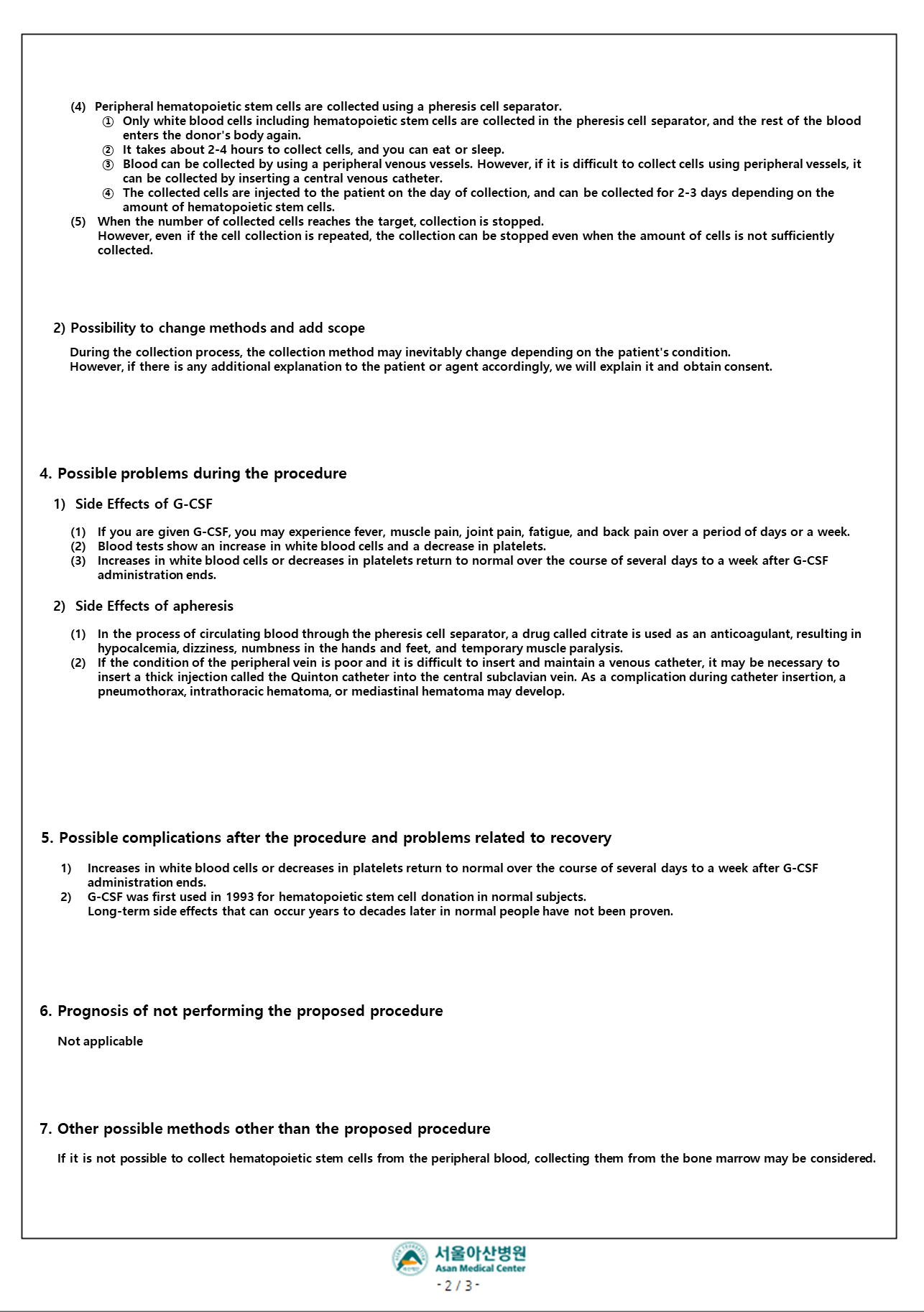

Supplement: S1 Data — (ZIP) [file pone.0246191.s001.zip › ConsentfromDonor2E.png]

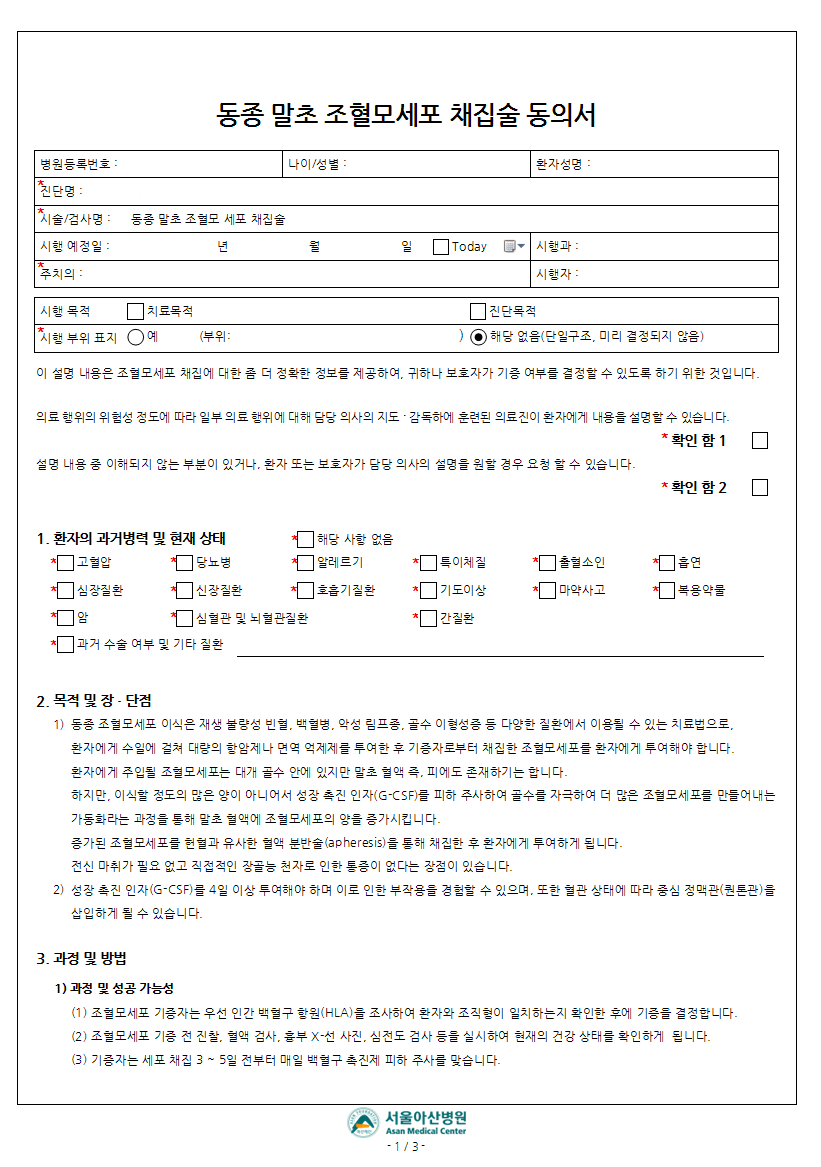

Supplement: S2 Data — (ZIP) [file pone.0246191.s002.zip › ConsentfromDonor1K.png]

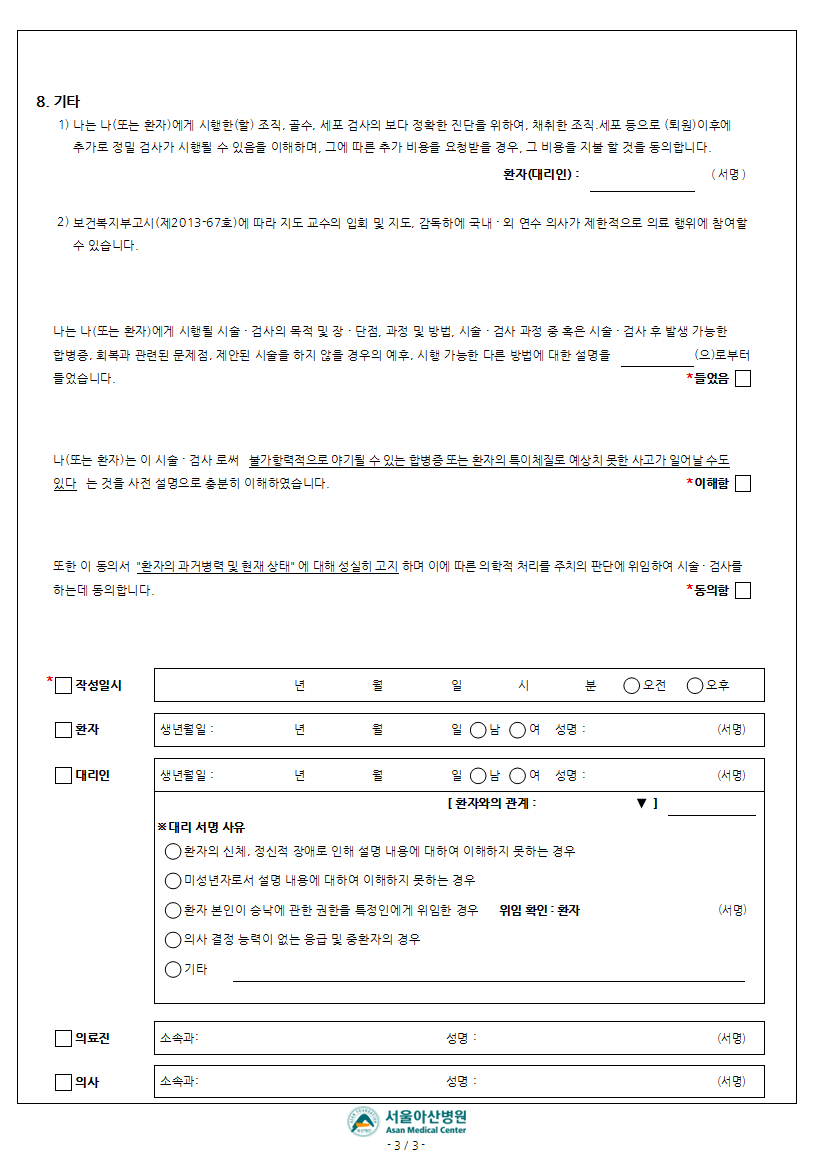

Supplement: S2 Data — (ZIP) [file pone.0246191.s002.zip › ConsentfromDonor3K.png]

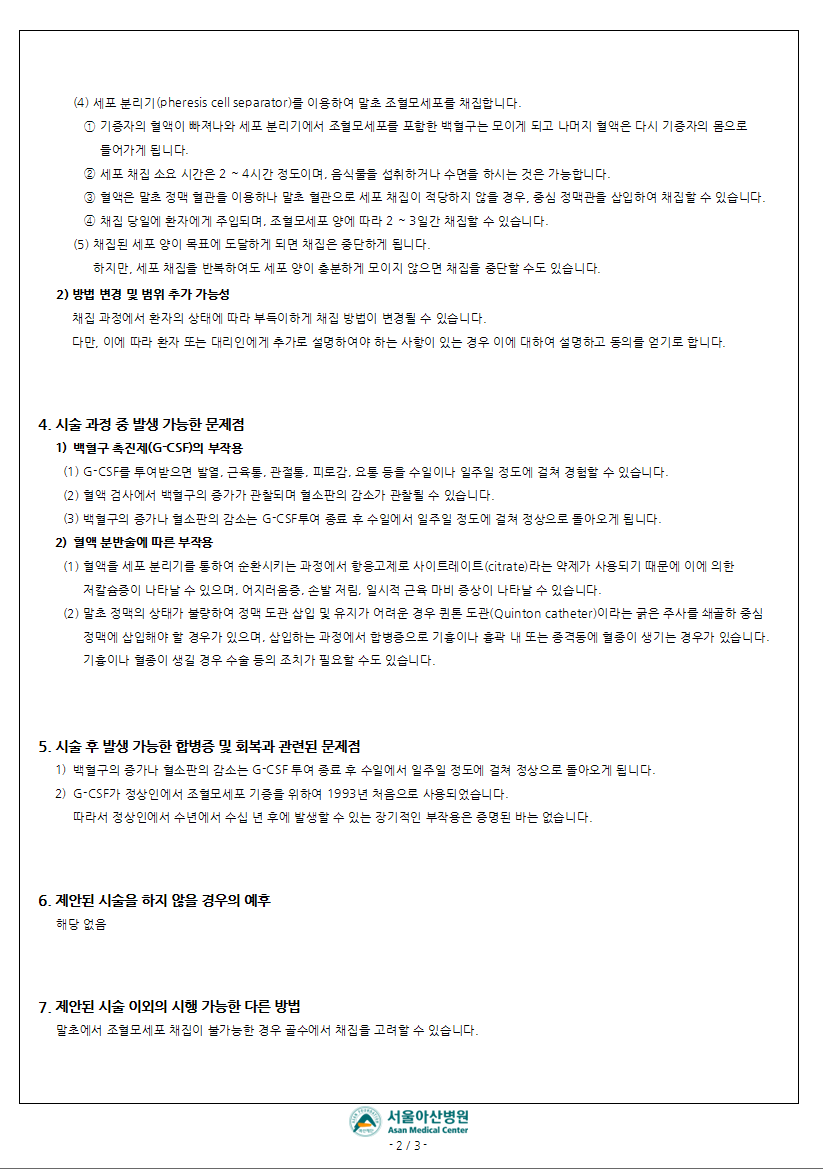

Supplement: S2 Data — (ZIP) [file pone.0246191.s002.zip › ConsentfromDonor2K.png]
